# Steroid receptor coactivators – their role in immunity

**DOI:** 10.3389/fimmu.2022.1079011

**Published:** 2022-12-13

**Authors:** Yosi Gilad, David M. Lonard, Bert W. O’Malley

**Affiliations:** ^1^ Department of Molecular and Cellular Biology, Baylor College of Medicine, Houston, TX, United States; ^2^ CoRegen, Inc., Baylor College of Medicine, Houston, TX, United States

**Keywords:** steroid receptor coactivators (SRCs), nuclear coactivators (NCoAs), nuclear factor-κB (NF-κB), inflammation, macrophages, Th17 cells, Treg cells

## Abstract

Steroid Receptor Coactivators (SRCs) are essential regulators of transcription with a wide range of impact on human physiology and pathology. In immunology, SRCs play multiple roles; they are involved in the regulation of nuclear factor-κB (NF-κB), macrophage (MΦ) activity, lymphoid cells proliferation, development and function, to name just a few. The three SRC family members, SRC-1, SRC-2 and SRC-3, can exert their immunological function either in an independent manner or act in synergy with each other. In certain biological contexts, one SRC family member can compensate for lack of activity of another member, while in other cases one SRC can exert a biological function that competes against the function of another family counterpart. In this review we illustrate the diverse biological functionality of the SRCs with regard to their role in immunity. In the light of recent development of SRC small molecule inhibitors and stimulators, we discuss their potential relevance as modulators of the immunological activity of the SRCs for therapeutic purposes.

## Introduction

Transcription is an essential biological process that lies at the intersection between the environment and the genome. Nuclear receptors (NRs) comprise a large family of transcription factors (TFs), which are prominent regulatory components of transcription (1–3). NRs bind directly to hormone-response elements on the DNA to mediate gene activation or repression. Recruitment of NRs to the DNA is a necessary, but not a sufficient step for transcription to commence. The most critical step in this process is the subsequent recruitment of another class of proteins to the DNA-bound NRs, called nuclear coactivators (NCoAs). The binding of NCoAs to a NR promotes the assembly of the transcriptional machinery and it is therefore a key step required for all transcription to ensue (4, 5). NCoAs are key regulators of transcription that are capable of interacting with a wide variety of NRs and other TFs, possessing pleiotropic effects that underlie their activity on human physiology and pathology. Steroid Receptor Coactivators (SRCs) are a family of three homologous NCoAs that are involved in the transcriptional activity of a majority of NRs. Since the discovery of the first SRC, SRC-1 (6), the ubiquitous expression of the SRCs and their critical impact on major physiological processes through their genome-wide activity has been revealed, with special attention paid to their roles in genetics, development, reproduction, metabolism and cancer biology, with an emphasis on hormone-related diseases (7, 8).

Despite the recognition that the SRCs play an important role in human immunology, mainly through fate-determination of immune cells and regulation of inflammatory processes, there is a paucity of reviews that summarize this aspect of their biology. Here we review the literature that establishes the SRCs as important regulators of immunity through their interactions with a major regulator of inflammation – nuclear factor-κB (NF-κB) as well as their emerging importance to the development and functionality of various immune cell types. On the basis of the current knowledge, we outline the potential of targeting the SRCs in immune cells as an approach to achieve improved clinical outcomes in cancer and autoimmune diseases.

## Nuclear receptors and SRCs – background

### In physiology

NRs are a large family of TFs that is comprised of 48 members in humans. NRs are defined by their ability to bind directly to *cis* regulatory elements on DNA to regulate gene expression (1–3). The biological activity of the majority of human NRs is ligand-dependent, while a dozen of them are considered “orphan” receptors, since they either have no cognate ligand or their ligands have not yet been identified (9). All NRs are unable by themselves to drive transcription, relying on another family of gene-regulator proteins, NCoAs (7, 8). NCoAs are critically important to major physiological processes including development, metabolism, homeostasis and immunity (10). NCoAs do not bind to DNA directly, but instead elicit their transcription regulatory roles by binding to NRs. Once bound to a NR NCoAs promote chromatin accessibility, inter alia due to their ability for covalent modification of histones (1), allowing for the subsequent recruitment of requisite components of the transcriptional machinery (5, 11, 12).

SRCs are a family of NCoAs comprised of three homologous p160 proteins; SRC-1 (NCoA1), SRC-2 (NCoA2/TIF2/GRIP1) and SRC-3 (NCoA3/AIB1/ACTR/TRAM1/RAC3), which are involved in the transcriptional activity of a majority of NRs and other TFs. SRCs are the most studied family of NCoAs, they are ubiquitously expressed and have a broad impact on essential physiological and pathological processes such as reproduction, metabolism, growth and development, genetic diseases, carcinogenesis and immunity (7, 8). All three members of the SRC family have a molecular weight of approximately 160 kDa, share a high degree of sequence similarity and all contain three main functional domains; a basic helix-loop-helix/Per/ARNT/Sim (bHLH-PAS) domain, a nuclear receptor interaction domain (RID) with two LXXLL motifs (X, any amino acid), and two C-terminal activation domains - AD1 and AD2 (7, 13). Since the discovery and identification of SRC-1 as the first NCoA in 1995 (6), and the subsequent discovery of the other two SRC family members, SRC-2 (14) and SRC-3 (15–19), more than 300 transcriptional coactivators have been reported in the literature (12). The biological importance of the SRCs encompasses a wide variety of essential physiological functions as evident from numerous research papers and review articles. Proper functioning of each one of the SRCs is critical for physiological homeostasis at both cellular and whole-organism levels. SRCs do not just activate genes randomly, but coordinately activate large numbers of genes for physiologic goals. For example, SRC-1 regulation of the estrogen receptor-α (ER) and progesterone receptor (PR) is critical for normal uterine development and function, and loss of SRC-1 results in partial resistance to hormone stimulation and impaired estrogen-induced uterine growth (20). SRC-1 is also important for the full transcriptional activity of peroxisome proliferator-activated receptor gamma (PPARγ) (21), a critical NR for adipose tissue development (22). Cooperative activity of SRC-1 and SRC-3 is required for the expression of genes that are essential for brown adipose tissue development and it was demonstrated that double SRC-1/SRC-3 knockout (KO) results in incapability of lipid storage and brings about defective thermogenesis (23). SRC-2 plays an essential role in the fertility of both male and female mice; SRC-2 gene deletion in male mice impairs spermatogenesis and brings about age-dependent testicular degeneration, while hypofertility in female SRC-2 null mice causes placental hypoplasia (24). Loss of SRC-2 in mice causes growth retardation and reduced adiposity (24), linking SRC-2 activity to two additional major physiological processes – development and metabolism. Further explorations revealed the central role that SRC-2 plays in metabolism, establishing it as a ‘master regulator’ of lipid metabolism (25–28). SCR-3 is a strong NCoA of ER and PR transcriptional activity (17, 29) and therefore is critical for normal mammary gland growth and development (30, 31). Recent cryo-electron microscopy (Cryo-EM) based studies have demonstrated that SRC-3 is recruited by its primary NR – ERα (32) as well as by AR (33), shedding light on the architecture of biologically active SRC-3 complexes and substantiating its regulatory role in transcription. Like the other two SRC family members, lack of SRC-3 also results in major, whole-body level physiological abnormalities; KO of SRC-3 in mice results in reproductive malfunction, dwarfism and delayed pubertal development (30).

### In cancer

All three SRCs are strongly associated with tumorigenic processes including metastasis, especially in hormone-related cancers (34). In breast cancer (BC) SRC-1 is an oncogene with specific roles in cell migration and metastasis (35, 36). The importance of SRC-1 to metastasis formation was demonstrated by a study that looked at inactivation of SRC-1 in a polyoma middle T (PyMT) BC mouse model; KO of SRC-1 did not affect tumor initiation, however it significantly reduced lung metastases (37). SRC-1 has an increased expression in HER-2 positive BC and is positively correlated with poor prognosis, resistance to endocrine therapy and recurrence (38, 39). SRC-1 was also linked to tumorigenesis in prostate, thyroid and endometrial cancers, which positions it as an important oncogene in hormone-related malignancies (40). SRC-2 is a NCoA of the androgen receptor (AR) and is highly associated with poor survival in prostate cancer (41, 42). Overexpression and amplification of SRC-2 were positively correlated with high tumor grade and poor survival (41, 43, 44). Moreover, it was shown that the importance of SRC-2 to the regulation of energy homeostasis and metabolism (27, 45–47) plays a primary role in prostate cancer cell survival and metastasis (26). The association of SRC-3 with tumorigenesis in various cancers has been demonstrated by multiple studies (17, 48–54), establishing it as pan-cancer oncogene. However, as is implied by its alias, amplified in breast cancer 1 (AIB1), SRC-3 is a driving force for tumorigenesis in ER positive BCs (17, 55, 56). Overexpression and amplification of SRC-3 in ER positive BCs is associated with the aggressiveness of the disease (55) and positively correlates with poor prognosis (57, 58). In two genetically engineered mouse models - Mouse Mammary Tumor Virus (MMTV)-v-ras and MMTV-Erbb2 - tumorigenesis and development of breast cancer were significantly reduced by inactivation of SRC-3 (59, 60). Mutations in the ligand binding domain of the *ESR1* gene lead to ligand-independent activation of mutant ER proteins, which also makes them resistant to anti-estrogen therapy. Unlike wild type (WT) ER, whose interaction with the SRCs is normally estrogen-dependent, mutant versions can interact with SRC-3 in an absence of a ligand (61, 62). Ligand-unregulated interaction of SRC-3 with mutant ER leads to constitutive oncogenic transcriptional activity, which exemplifies the unique and critical role of SRC-3 as an oncoprotein in ER positive BCs.

## Coactivators and the immune system

### Interaction with NF-κB

**Figure 1:**
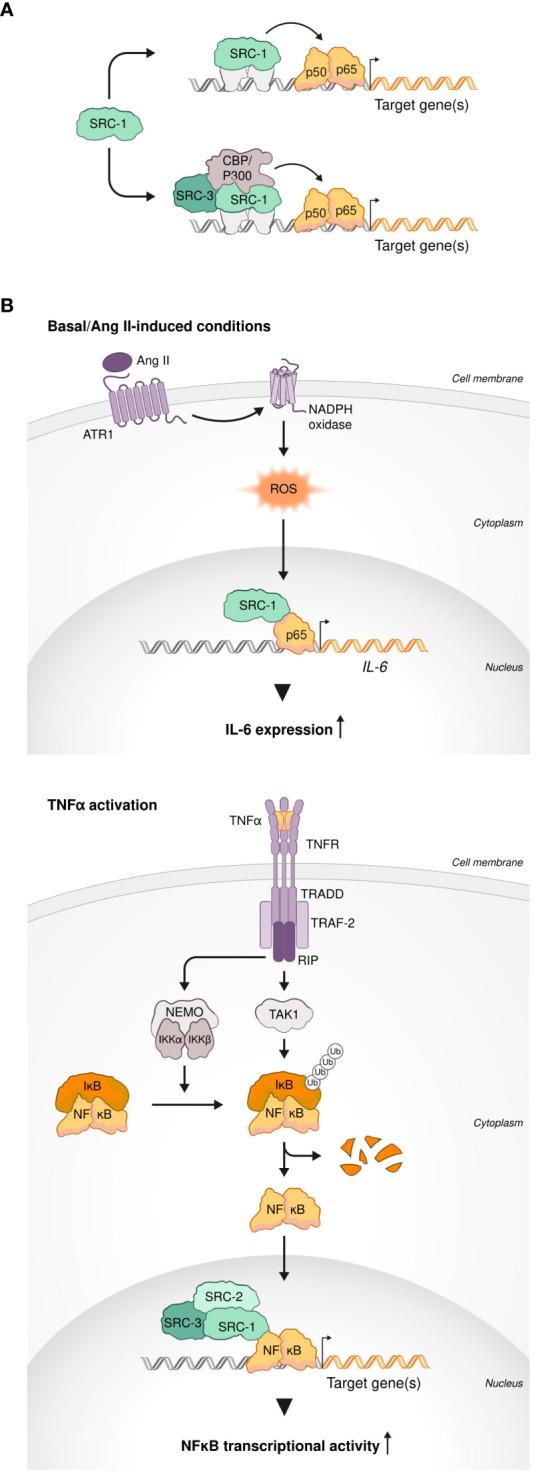
SRCs interactions with NF-κb. **(A)** SRCs are NF-κB coactivators. SRC-1 can coactivate NF-κB transcriptional activity in an independent manner (top). SRC-1 and SRC-3 cooperate with other coactivators (CBP and p300) for synergistic coactivation of NF-κB (bottom). **(B)** SRCs drive transcriptional activity of NF-κB under inflammatory-like conditions. SRC-1 interacts with p65 to induce *IL-6* transcription under basal and angiotensin II (Ang II)-induced conditions (top). Under TNFα activation, each member of the SRC family (SRC-1, SRC-2 and SRC-3) can drive NF-κB transcriptional activity (bottom).

Nuclear factor-κB (NF-κB) is a family of five DNA-binding nuclear factors that form a variety of homo- and hetero-dimers (63). NF-κB dimers exist in the cytoplasm in a form of an inactive complex with one of the three inhibitor of NF-kB (IκB) proteins. NF-κB activation takes place when stimulatory signals bring about phosphorylation of IκB by the IκB kinase (IKK) complex and the consequent ubiquitination and proteasomal degradation of IκB. Release from the complex with IκB allows the translocation of NF-κB into the nucleus and initiation of its transcriptional activity (64). Although NF-κB is important for many physiological processes (65), it is predominantly associated with inflammation and regulation of innate host defense (64). Consistent with the centrality of SRCs to various fundamental physiological processes, their involvement in both immune-related and general NF-κB transcriptional activities has been well established; SRC-1 was the first SRC family member whose selective interaction with the NF-κB subunit p50 was demonstrated by yeast two-hybrid and Glutathione S-transferase (GST) pull down assays (66). The authors of this study also showed that SRC-1 can mediate NF-κB transcriptional activity either independently or in cooperation with CBP or p300 to achieve a synergized coactivation of NF-κB (**Figure 1A**). Synergistic coactivation of NF-κB by CBP was also observed with overexpression of SRC-3, as indicated by Rel-A-Luc reporter assay in HeLa cells (67), reflecting a functional overlap that is not uncommon between the SRC family members (8). Overexpression of either SRC-2 or SRC-3 was positively correlated with p65-associated IκB-luc reporter activity in a dose-dependent manner. This observation was consistent with a decrease of IκBα protein levels following SRC-2 or SRC-3 knock down (KD), suggesting a functional overlap in the regulation of NF-κB also between SRC-2 and SRC-3 (68). Intra-nuclei microinjection of specific anti-CBP/SRC-1/pCAF blocking antibodies in Rat-1 cells demonstrated that all three SRCs are indispensable for p65-dependent gene expression (69). Implementing a CBP mutant that is lacking the SRC-1 interacting domain, the authors of this study also showed that p65-mediated gene expression is dependent upon SRC-1-CBP interaction. Consistent with that, mutation in LXD4, the LXXLL CBP-recognition domain of SRC-1 (70) resulted in failure to rescue NF-κB-dependent gene expression under conditions of SRC-1 blockade. Interaction of SRC-1 with p50/p65 drives the expression of Vascular Endothelial Growth Factor C (VEGFC) in a thyroid carcinoma cell line TPC-1, while KD of SRC-1 results in a decrease of VEGFC transcription and expression. SRC-1 KD-associated decrease of VEGFC levels correlated with reduced lymphangiogenesis *in vivo*, which represents physiological implications of SRC-1-mediated coactivation of NF-κB (71). Evidences of physical interactions between NF-κB family members and all three SRCs as well as participation of the SRC family members in transcriptional complexes with other coactivators to drive NF-κB activity, establish the SRCs as important transcriptional partners of NF-κB and implies their direct involvement in the regulation of immunity.

Indeed, in a study that have tested four different coactivators (CBP, p300, P/CAF and SRC-1), SRC-1 most effectively cooperated with p65 to induce transcriptional activation of the promoter of a proinflammatory gene *IL-6* under basal and angiotensin II (Ang II)-induced conditions in CHO-AT_1a_ cells (72). Using rat vascular smooth muscle cells (rVSMC) as an experimental model, the authors demonstrated that Ang II-induced activation of the *IL-6* promoter is regulated by ERK-mediated phosphorylation of SRC-1 (**Figure 1B**), shedding light on the molecular mechanism of this process. In agreement with the observation that overexpression of either SRC-1 or SRC-2 increases p65-dependent transcription (69), it was demonstrated that under inflammatory-like conditions, mimicked by Tumor necrosis factor alpha (TNFα) activation, both NCoAs can drive NF-κB transcriptional activity. Moreover, SRC-1 substituted SRC-2 in the IκBα promoter of HEK293 cells to allow robust NF-κB-mediated transcription (68). TNFα-induced NF-κB activity was enhanced by the third p160 family member – SRC-3 - as well in a dose dependent manner as was demonstrated using HeLa cells (**Figure 1B**) (67). This observation was further supported by the evidence of physical interaction between SRC-3 and p65 (Rel-A) provided by co-immunoprecipitation experiments. Furthermore, an increase in the NF-κB transcriptional activity under TNFα stimulation was positively correlated with SRC-3 phosphorylation, possibly by IKK, and its accumulation in the nucleus (73), which provides an insight into the molecular mechanism of SRC-3-mediated regulation of NF-κB. Interestingly, SRC-3 deficiency results in an IKK-dependent increase in lymphocytes numbers that is specific to lymphoid organs. This observation correlates with a selective increase of phosphorylated IκB as well as nuclear translocation and transcriptional activity of NF-κB in the thymus, bone marrow (BM) and spleen (74). Collectively these data suggest that through physical interaction with IKK, in a context dependent manner, SRC-3 can either act as a driving force of NF-κB-mediated transcription or, by limiting the ability of IKK to phosphorylate IκB, exert an inhibitory function on it. The capability of the SRCs to mediate both transcriptional activation and repression, as a variable of a specific biological context and post-translational modification events, is evident from additional studies (13). This reflects the wide scope of the biological functionality of the SRC family members and specifically underscores the importance of SRC-3 to immunity as a bi-directional regulator (activator or repressor) of NF-κB. Overall, the solid connection between the SRCs and NF-κB mediated transcriptional activity that reflects, especially but not exclusively, in the expression of a prominent inflammatory gene - *IL-6* – under either basal or inflammatory-like conditions, suggests a primary role of the SRCs in key immunological processes. However, more direct studies are yet required for corroborating a direct connection between the parts in the ‘SRC-NF-κB-immunity’ axis.

### SRC-2 and SRC-3 in macrophages

**Figure 2:**
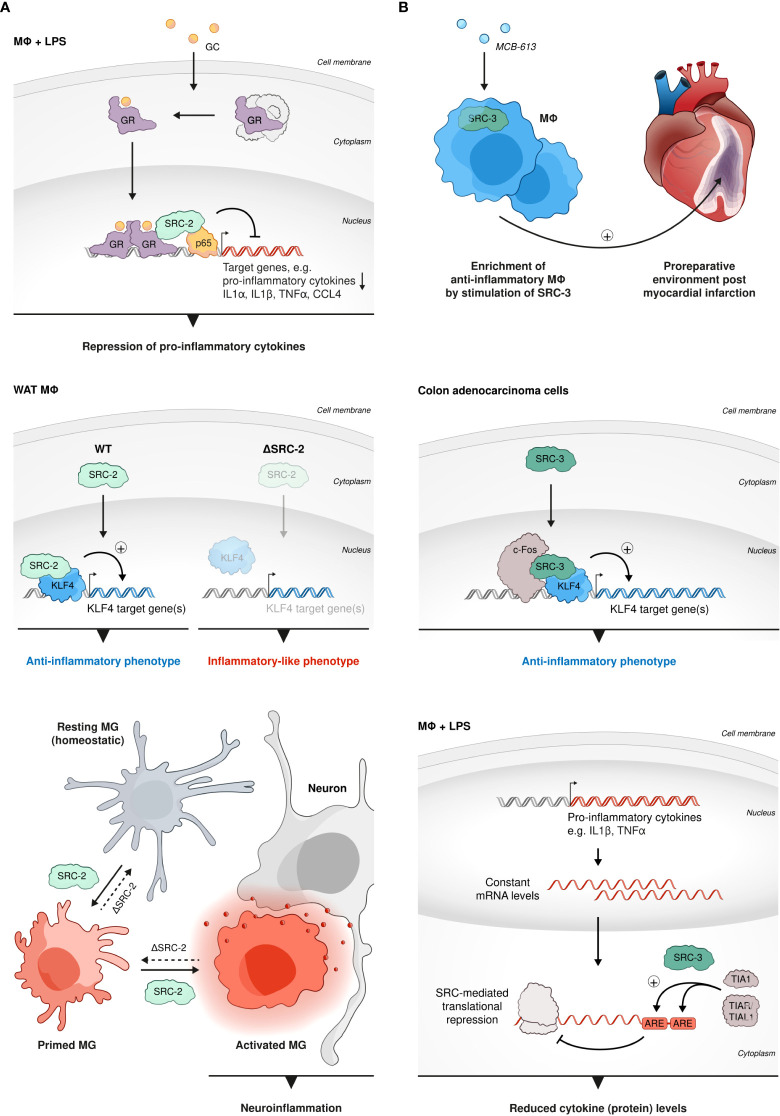
SRCs role in MΦs. **(A)** Anti-inflammatory and pro-inflammatory roles of SRC-2 in MΦs. SRC-2 participates in GC-mediated repression of pro-inflammatory cytokine genes: GR-mediated repression of *IL1α*, *IL1β*, *TNFα*, and *CCL4* in BMMΦs, following LPS stimulation, positively correlates with co-recruitment of GR and SRC-2 to NF-κB-binding sites occupied by p65 (top). SRC-2 is recruited along with KLF4 to KLF4 target genes in WAT MΦs to promote the induction of anti-inflammatory commitment of these cells (middle left). SRC-2 KO results in attenuated KLF4 expression and activity and drives the shift of WAT-resident MΦs into an inflammatory-like phenotype (middle right). In MG SRC-2 drives neuroinflammation through activation of a proinflammatory program which reflects in a homeostatic signature of MG cells and reduced EAE severity in mice with conditional SRC-2 KO in myeloid cells (bottom). **(B)** SRC-3 has an anti-inflammatory role in MΦs. SRC-3 stimulation brings about enrichment of anti-inflammatory MΦs: Anti-inflammatory MΦs are enriched by stimulation of SRC-3 with a small molecule stimulator *MCB-613* to promote the establishment and maintenance of a pro-reparative environment post MI (top). In colon adenocarcinoma cells SRC-3 interacts with c-Fos to promote KLF4-related gene expression to regulate inflammation (middle). SRC-3 downregulates proinflammatory cytokines: following LPS-stimulation SRC-3 downregulates protein levels, but not mRNA amounts, of proinflammatory cytokines such as TNFα and IL-1β in MΦs, which indicates translational derepression rather than GR-related transcriptional suppression (bottom).

Macrophages (MΦs) have a ubiquitous presence in all parts of the body and they represent a key component in diverse physiological processes such as development, tissue remodeling and metabolism, to name just a few (75, 76). However, the most important activities of MΦs are related to host defense and orchestration of inflammatory processes (77, 78). SRC-regulated transcriptional activity has a crucial impact on the functioning of MΦs, which particularly, but not exclusively reflects, in MΦs’ immunomodulatory activity: Glucocorticoid (GC) receptor (GR) is an important restrainer of inflammation (79). GC-mediated repression of pro-inflammatory cytokine genes such as *IL1α*, *IL1β*, *TNFα*, and *CCL4* in BMMΦs following lipopolysaccharide (LPS) stimulation, positively correlates with co-recruitment of GR and SRC-2 to NF-κB-binding sites occupied by p65 (80) (**Figure 2A**). Induction of these canonical pro-inflammatory genes by either LPS, a Toll like receptor (TLR) 4 ligand, or other TLR ligands, such as Pam3Cys or CL264 (TLR2 and TLR7 ligands), was reduced by stimulation of GR activity with dexamethasone (Dex) in SRC-2 KO but not WT MΦs. Moreover, transcriptome analysis revealed that SRC-2 has a much broader impact on GR-mediated repression of inflammatory genes showing that over 60 out of 152 LPS induced Dex-repressed genes were derepressed in SRC-2 KD but not WT BMMΦs. Consistent with these *in vitro* observations, the authors also showed that SRC-2 KO in MΦs increased mice susceptibility to LPS-induced endotoxin shock *in vivo*, demonstrating the physiological significance of SRC-2-related regulation of MΦ function. Further support to the above observations was provided using MΦ-like RAW264.7 cells where GR activation-dependent downregulation of IRF3 pro-inflammatory target genes expression was substantiated by overexpression of SRC-2 (81). Consistent with the anti-inflammatory role of SRC-2, as a NCoA of GR-mediated repression of pro-inflammatory cytokines in BMMΦs, under a prolonged high fat diet (HFD) regime and subsequent recruitment of monocytes into fat pads, SRC-2 deficiency predisposes mice to adipose tissue inflammation, which is accompanied by increased transcript levels of pro-inflammatory genes (82). Though the function of SRC-2 and its physiological importance as a GR corepressor in pro-inflammatory MΦs is evident from these observations, further research is needed to shed more light on the molecular mechanism of this SRC-2-mediated GR activity. Another important function of SRC-2 in MΦs is associated with its capability to coactivate an essential regulator of MΦ polarization, KLF4 (83); SRC-2 is recruited along with KLF4 to KLF4 target genes in white adipose tissue (WAT) MΦs to promote the induction of an anti-inflammatory commitment of these cells (**Figure 2A**). Furthermore, SRC-2 KO that results in an attenuated KLF4 expression and activity, drives the shift of WAT-resident MΦs into an inflammatory-like phenotype. Intriguingly, in a different experimental system it has been shown that SRC-3 interacts with c-Fos to promote KLF4 expression in colon adenocarcinoma cells and that the loss of SRC-3 is associated with colon inflammation *in vivo* (84) (**Figure 2B**). In agreement with this observation, stimulation of SRC-3 with a small molecule stimulator MCB-613 (85) brings about enrichment of anti-inflammatory MΦs to promote the establishment and maintenance of a pro-reparative environment post myocardial infarction (MI) (86) (**Figure 2B**). Surprisingly, as opposed to its anti-inflammatory role in peripheral MΦs, SRC-2 drives neuroinflammation through activation of a proinflammatory program in microglia (MG) (87); conditional KO (cKO) of SRC-2 in myeloid cells significantly reduces experimental autoimmune encephalomyelitis (EAE) severity, which in part is associated with persistence of a homeostatic MG signature, rather than a pro-inflammatory profile and activity that are associated with SRC-2 deficiency in BM and WAT-resident MΦs (**Figure 2A**). This unexpected role of SRC-2 as a driving force of neuroinflammation indicates the functional versatility associated with this NCoA in different MΦ subtypes which is dependent on the environmental conditions and biological context. Collectively, these studies demonstrate that SRC-2 plays an important, multi-functional role in activation and function of different types of MΦs. Interestingly, like SRC-2, SRC-3 is also capable of downregulating the proinflammatory cytokines TNFα and IL-1β in MΦs in cell culture, however through derepression of translation rather than through GR-related transcriptional suppression (88); following LPS-stimulation, elevated protein levels of TNFα, IL-6, and IL-1β were detected in SRC-3 KO MΦs as compared to WT controls. However, mRNA amounts of the relevant genes remained unchanged between SRC-3 KO and control MΦs, which infers that SRC-3-mediated repression of pro-inflammatory cytokines occurs on a translational, rather than transcriptional level and is not associated with GR activity. It has been shown that SRC-3 promotes the binding of translational repressors such as TIA1 and TIAR/TIAL1 to adenylate and uridylate (AU)-rich elements (AREs) in the 3’-untranslated region (UTR) of the mRNA transcript of the TNF gene, which sheds light on the molecular mechanism that underlies the indicated translational repression (**Figure 2B**). Indeed, SRC-3 KO MΦs produce enhanced levels of TNFα protein in response to LPS stimulation, but without affecting the mRNA levels of the *TNFα* gene or genes of other related pro-inflammatory cytokines such as *IL-6*, and *IL-1β*. Consistent with these observations, the protein levels of TNFα, IL-6, and IL-1β were elevated as a result of *E. coli*-induced peritonitis in SRC-3 KO mice (89). Counterintuitively, despite the elevation of pro-inflammatory cytokines, these mice failed in bacterial clearance. Apparently, loss of SRC-3 in MΦs results in incapacity to produce catalase, a key antioxidant enzyme that reduces reactive oxygen species (ROS), which consequently sensitizes MΦs to apoptosis. Additionally, SRC-3 deficiency decreases expression of SR-A protein, which is an important component for Fc-independent phagocytosis function of MΦs. Altogether these observations highlight the importance of SRC-3 in regulation of MΦ-mediated inflammation as well as their proper phagocytic activity and viability *in-vivo*. In summary, both SRC-2 and SRC-3 have a broad impact on MΦ function that is highly dependent on the biological context and MΦ type and exemplifies the functional plasticity of the SRCs. Data from mouse models indicate that SRC-2 and SRC-3 are both important for restraining cytokine storms and severe immuno-toxic effects, which underscores their physiological significance.

### SRC-1 and SRC-3 in Th17 cell development and function

**Figure 3:**
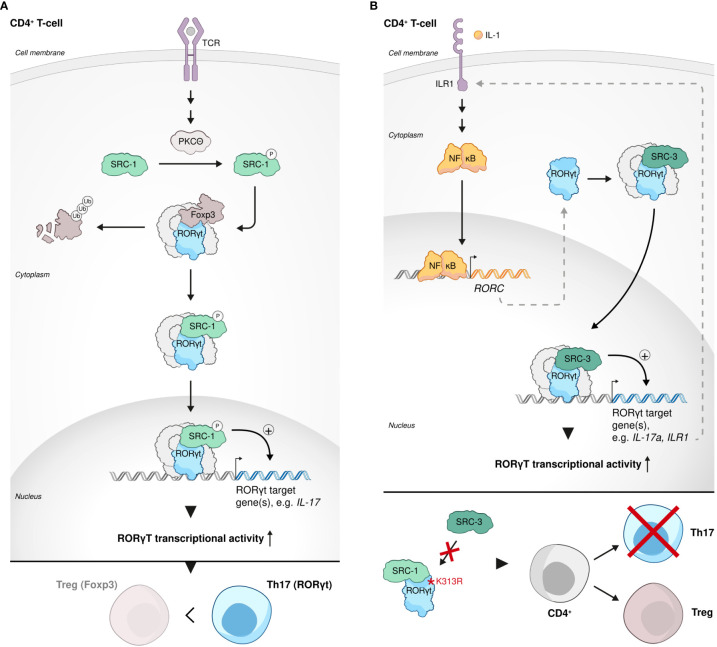
SRCs in Th17 cell development and function. **(A)** SRC-1-RORγt complex stimulates phenotypic dominance of the Th17 cell lineage over the Treg cell lineage. Following phosphorylation by protein Kinase C θ (PKCθ), SRC-1 replaces FOXP3 in its complex with RORγt in CD4+ cells, subjecting FOXP3 to proteasomal degradation and stimulating Th17 cell lineage phenotypic dominance. **(B)** SRC-3 is an essential regulator of Th17 cell genes. SRC-3 regulates pathogenic inflammation *via* coactivation of RORγt-associated expression of Th17 cell genes through an IL-1-ILR1 mediated signaling axis (top). Interaction of RORγt with SRC-3 is important for healthy Th17 cell lineage development: a K313R mutation of RORγt specifically disrupts the ability of RORγt to interact with SRC-3 but not SRC-1 and impairs healthy differentiation and development of Th17 cells (bottom).

T helper 17 (Th17) cells are a subtype of CD4 cells, whose primary physiological function is related to clearance of extracellular pathogens such as bacteria and fungi (90). However, Th17 cells are best known as pathogenic drivers of multiple autoimmune-related diseases, including psoriasis, rheumatoid arthritis (RA), inflammatory bowel disease (IBD), Crohn’s disease and multiple sclerosis (MS). IL17A is the hallmark cytokine of Th17 cells and the primary cytokine that is associated with Th17 cell-mediated autoimmunity (91). Regulatory T cells (Tregs) are characterized by the expression of the TF forkhead box protein P3 (*FOXP3*), which determines their genetic and epigenetic signatures and lineage commitment (92). The canonical biological function of Tregs is anti-inflammatory in its nature and it is shaped to restrain an excessive activity of effector T cells, e.g. Th17 cells, and by that means to ensure immune homeostasis and prevent autoimmune disorders (93, 94). Intriguingly, the anti-inflammatory Tregs and pro-inflammatory Th17 cells that usually antagonize each other’s function, equally require Transforming growth factor beta (TGFβ) signaling at their early development for the establishment of transcription factor networks that shape their lineage commitment (95, 96). Treg vs Th17 fate decision in the presence of TGFβ is governed by the cytokine milieu that tips the balance of the competitive antagonism between FOXP3 and ROR- family members TFs in the developing CD4 cell. Moreover, under certain conditions, Tregs can be re-differentiated into effector Th17-like cells with downregulated expression of *FOXP3* and ability to produce IL17A (95, 96). In the context of the demonstrated plasticity of the Treg-Th17 lineage barrier and the central but opposing roles of both these CD4 subsets in immunity, we mention in the following section the participation of SRC-1 in the regulation of the intimate interplay between the Treg and Th17 cell programs as it reflects by a SRC-related transcriptional regulation of RORγt: While both retinoic acid-related orphan receptors (RORs) RORγt and RORα are required for optimal Th17 cells development and function, RORγt is considered the master regulator of Th17 cell differentiation and IL17A production (97). Following phosphorylation by protein Kinase C θ (PKCθ), SRC-1 replaces FOXP3 in its complex with RORγt in CD4+ cells, which subjects the released FOXP3 to proteasomal degradation. At the same time, the resulting SRC-1-RORγt complex stimulates RORγt transcriptional activity, leading to phenotypic dominance of the Th17- over Treg-cell lineage (98) (**Figure 3A**). Mediation of PKCθ-dependent regulation of Th17, suggests that SRC-1 integrates TCR-signaling to induce a RORγt transcriptional program and Th17 differentiation. Alternately, it was shown that SRC-3 regulates pathogenic inflammation *via* coactivation of RORγt-associated expression of Th17 genes through an IL-1-ILR1 mediated signaling axis (99) (**Figure 3B**). Importantly, in an EAE mice model, whole body KO of SRC-1 (98) or cKO of SRC-3 in CD4 cells (99) resulted in resistance to autoimmune pathogenesis. These observations underscore the physiological importance of the orchestrated transcriptional regulation mediated by SRC-1 and SRC-3, which eventually dictates the fate and functionality of Th17 cells. Another study demonstrated that a K313R mutation of RORγt, which specifically disrupts the ability of RORγt to interact with SRC-3 but not SRC-1, selectively impairs healthy differentiation of Th17 without impairing thymocytes development, exemplifying the essentiality of RORγt-SRC-3 interaction for healthy Th17 development (100) (**Figure 3B**). Interestingly, it has been shown that RORγt relies on its AF2 domain to recruit SRC-1 and SRC-2 through interaction with the NCoAs’ LXXLL motifs (101). Recruitment of SRCs by RORγt enhances its transcriptional activity and is required for thymocytes survival, possibly through RORγt-related indirect regulation of the anti-apoptotic Bcl-xl expression (101). Collectively these studies demonstrate the importance of all three SRC family coactivators to both the development and functional control of Th17 cells.

### SRC-3 role in lymphocyte proliferation, B-cell transcriptional regulation and NK cells effector function

**Figure 4:**
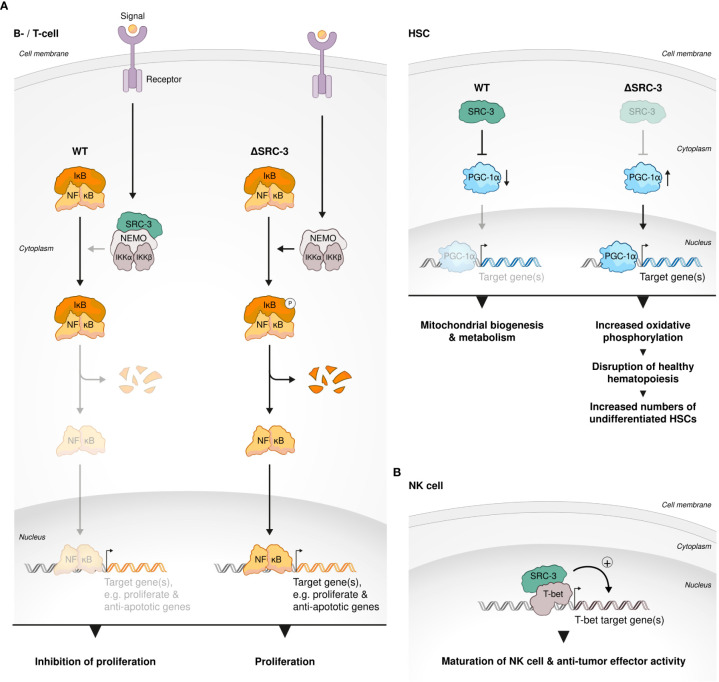
SRC-3 role in lymphocyte development. **(A)** SRC-3 plays an anti-proliferative role in early developing immune cells. SRC-3 selectively inhibits the proliferation of lymphoid cells in a cell autonomous manner: T and B cells isolated from SRC-3 KO mice showed an increased *ex vivo* proliferation compared to their WT counterparts (left). SRC-3 is required for maintaining the quiescent state of HSCs: lack of SRC-3 results in increased proliferation of HSCs. SRC-3 deficiency in murine HSCs results in an upregulated activity of PGC-1α (the master regulator of mitochondrial biogenesis and metabolism) an consequent disruption of normal HSC function and healthy hematopoiesis (right). **(B)** SRC-3 is important for healthy development of HSCs, NK and Treg cells. SRC-3 is a critical factor in lineage development and effector function of NK cells: SRC-3 is recruited to the promoter regions of prominent T-bet binding sites in WT but not SRC-3 KO NK cells.

SCR-3 has an established regulatory role in lymphoproliferation: Contrary to its proliferative role in cancer, SRC-3 selectively inhibits the proliferation of lymphoid cells, allegedly through its physical interaction with IKK, which prevents the phosphorylation of IκB that then inhibits NF-κB-mediated proliferative and anti–apoptotic genes (74). T and B cells isolated from SRC-3 KO mice showed an increased *ex vivo* proliferation compared to their WT counterparts, indicating a cell autonomous effect of SRC-3 on lymphocyte proliferation (74) (**Figure 4A**). Consistent with a key role of the SRCs in regulation of metabolism (27), in murine hematopoietic stem cells (HSCs) SRC-3 is highly expressed and its deficiency brings about upregulation of target genes of the master regulator of mitochondrial biogenesis and metabolism - Peroxisome proliferator-activated receptor-gamma coactivator (PGC-1α) (102). Upregulation of the PGC-1α transcriptional profile results in increased activity of oxidative phosphorylation in the mitochondria of HSCs and consequent disruption of normal HSC function and healthy hematopoiesis (103). Furthermore, regulation of mitochondrial metabolism in HSCs by SRC-3 is required for maintaining the quiescent state of these cells and lack of SRC-3 results in their increased proliferation (**Figure 4A**). This observation underscores the unexpected but critical role of SRC-3 as anti-proliferative factor in early developing immune cells. In agreement with the observation that SRC-3 is highly expressed in HSCs (103), it has been shown that SCR-3 levels in the thymus, which mainly contains pre-mature T cells, are elevated when compared with secondary lymph nodes (104). In murine B cell lymphocytes, SRC-3 (pCIP) was upregulated *via* IL-4-STAT6 axis (105). In a following publication the authors have suggested that an upregulation of SRC-3 by STAT6 is one part in a positive feedback loop of STAT6-mediated transcription (106). Given the centrality of STAT-6 in the B cell fate, activation, and function (106), these studies are extending and solidifying the overall importance of SRC-3 in transcriptional regulation and fate determination of lymphocytes.

In natural killer (NK) cells SRC-3 deficiency results in a decreased expression of key T-bet target genes such as *Zeb2*, *Prdm1*, and *S1pr5*, leading to compromised cell maturation, anti-tumor activity and signature phenotype (107). For example, key cytotoxic molecules, granzyme B and perforin as well as Interferon gamma (IFNg) production were reduced in SRC-3–deficient NK cells and *in-vivo* tumor surveillance of these cells was impaired as demonstrated using B16F10 mice melanoma model. Mechanistically, by a chromatin immunoprecipitation (ChIP)-sequencing experiment the authors have showed that SRC-3 is recruited to the promoter regions of prominent T-bet binding sites in WT but not SRC-3 KO NK cells (**Figure 4B**) only when T-bet is present, which suggests that SRC-3 acts as a coactivator of T-bet - a master TF in NK cells - and hence regulates their maturation, phenotype and activity.”

### SRCs role in Treg cell biology

**Figure 5:**
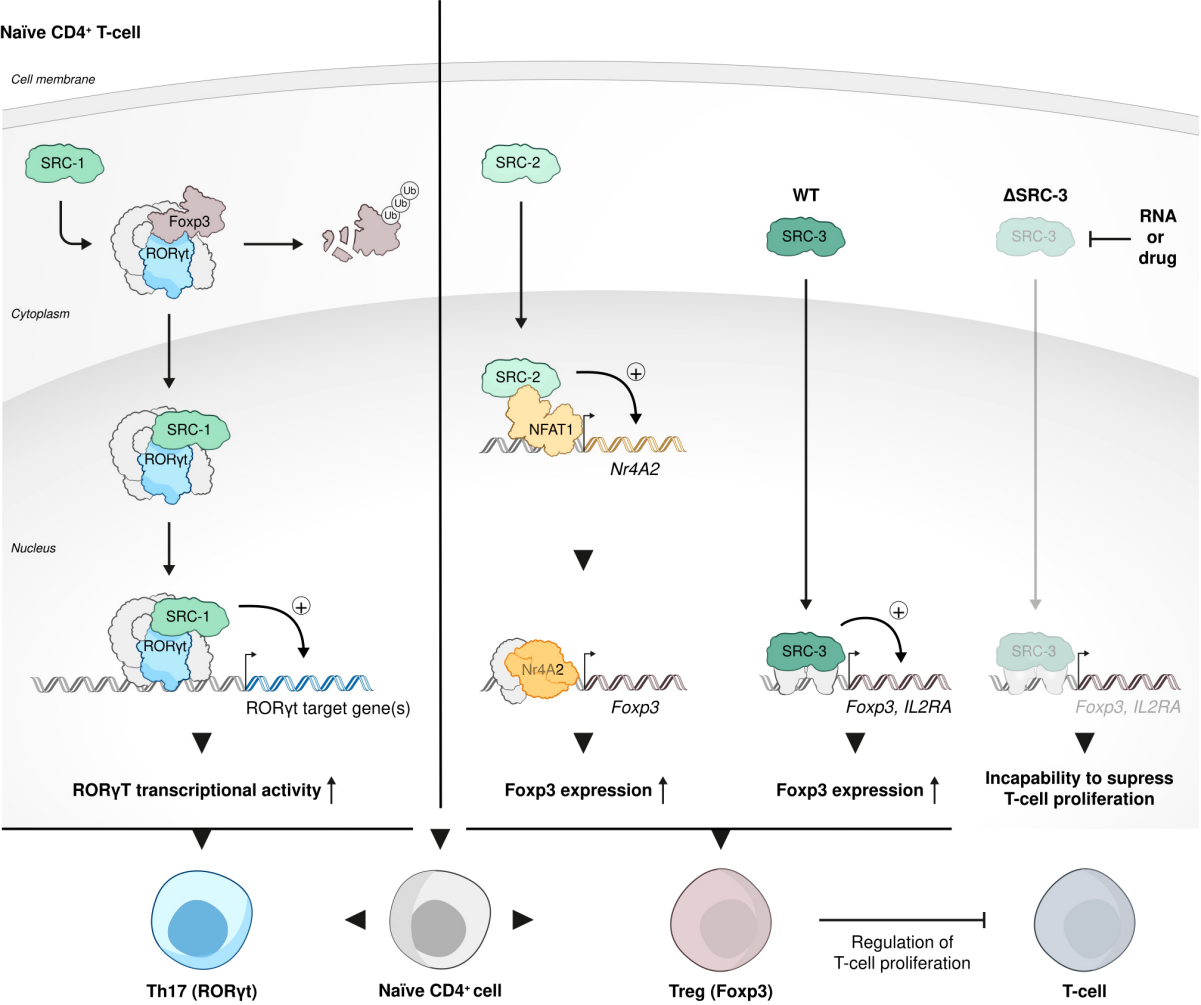
SRC-3 role in Treg cell biology function. Each one of the three SRCs has a distinct role in Treg cell biology and function. SRC-1 replaces FOXP3 in its interaction with RORγt to shift phenotypic dominance of CD4 cells from Treg cell to Th17 cells; SRC-2 stimulates Treg cell differentiation from naïve CD4+ T cells by coactivating NFAT1 to force the expression of a FOXP3 regulator Nr4a2; SRC-3 is enriched and critically important to the biological function of Tregs: inhibition of SRC-3 results in a decrease of transcript levels of the signature Treg cells genes - FOXP3 and IL2RA and consequent incapability of these Tregs to suppress proliferation of conventional T cells.

Interrogation of publicly available databases and direct cell-based assays have shown that like in HSCs, SRC-3 is enriched and critically important to the biological function of Tregs. *Ex vivo* experiments with human T cells have demonstrated that loss of SRC-3 function, achieved by RNA perturbation, and even more so by pharmacological inhibition, leads to a decrease in transcript levels of *FOXP3* and *IL2RA* - known Treg signature genes The loss of Treg signature genes brought about loss of the hallmark Treg phenotype - the ability to suppress proliferation of conventional T cells. Moreover, when subjected to pharmacological inhibition of SCR-3, resting CD4+CD30- T cells failed to acquire a Treg-like phenotype and function by exposure to Treg-inducing conditions *in vitro* (104) (**Figure 5**).

Intriguingly, in contrast to the phenotypic dominance of Th17 cells over Tregs that is conferred by SRC-1 (98), in a recently published study it has been shown that SRC-2 stimulates Treg differentiation from naïve CD4+ T cells *in vitro* and *in vivo* by coactivating NFAT1 to drive the expression of the FOXP3 regulator Nr4a2 (108). Using an EAE animal model, the authors of this study have demonstrated that SRC-2 KO mice are predisposed to severe autoimmune disorders compared to their counterparts with intact expression of SRC-2, exemplifying the essentiality of SRC-2 for maintaining immune-tolerance. Collectively, the studies mentioned above reflect a broad spectrum of biological functions that the SRCs play in Tregs, with SRC- 1 that is responsible for shifting the dominance from Tregs to Th17 cells within the CD4+ cells population (**Figure 3A**), while SRC-2 and SRC-3 play a Treg-phenotype supportive role (**Figure 5**).

## Conclusions and furtherer perspectives

SRCs are important regulators of gene expression with a broad impact on human physiology and pathology. Since the discovery of SRC-1 and the subsequent characterization of SRC-2 and SRC-3, almost three decades ago, SRC biology was a focus of intense scientific research. Involvement of the SRCs in the regulation of various aspects of immunity is well established in the literature. In this review we summarized the different roles of the SRCs as regulators of immunity through a description of their interaction with the major regulator of inflammation – NF-κB and their role in the development and function of different sub-sets of immune cells. All three SRCs can physically interact with and drive the transcriptional activity of NF-κB family members. Furthermore, several studies have shown a direct impact of SRC-3-driven NF-kB activity on the expression of a canonical pro-inflammatory gene *IL-6* (68, 109). Overall, the involvement of the SRCs in the regulation of NF-kB establishes them as important players in inflammatory processes. However, most of the observations that support SRC-mediated NF-kB activity are based on reporter assays. Hence, there is a need in additional studies to shed more light on the importance of SRC-regulated pro-inflammatory activity of NF-κB in the physiological context. Moreover, a very well-known phenomena of overlapping biological functions between the SRCs (27) also applies to their role in regulation of NF-κB. Therefore, more detailed understanding of the unique role of each SRC in the regulation of NF-κB activity in a given biological context is valuable from a fundamental scientific perspective and significant for translational purposes.

In different types of immune cells the SRCs are involved in essential processes that include fate-determination, development and function. In MΦs SRC-2 plays a multi-functional role and is capable to participate in two different mechanisms to exert an anti-inflammatory function; in a ligand-dependent manner SRC-2 is recruited by GR to facilitate GR-mediated repression of immediate-early (IE) proinflammatory genes in BMMΦs. In tissue resident MΦs SRC-2 operates in a GR-independent manner, as a coactivator of KLF4, to enforce homeostatic transcription program. As opposed to its anti-inflammatory functions in monocyte derived MΦs (e.g. BMMΦs and tissue resident MΦs), SRC-2 promotes neuroinflammation through activation of a proinflammatory program in MG (87). This surprising pro-inflammatory role of SRC-2 indicates its broad functional capacity in MΦs, which strongly depends on the type of the MΦs and their biological context. SRC-3 also plays an anti-inflammatory role in MΦs through downregulation of proinflammatory cytokines. However, a SRC-3-mediated anti-inflammatory program in MΦs relies on translational derepression rather than transcriptional suppression (88). Despite the disparity in the molecular mechanisms by which SRC-2 and SRC-3 mediate their anti-inflammatory program in MΦs, this represents another example of a functional overlap between p160 members. In addition to its anti-inflammatory role in MΦs, SRC-3-mediated regulation of reactive oxygen species (ROS) levels and production of SR-A protein are essential for MΦ phagocytic activity and viability. Overall these data represent high degree of functional diversity of SRC-2 and SRC-3 in MΦ biology.

In CD4+ Th17 cells, SRC-1 and SRC-3 can both interact with the major Th17 TF RORγt to shape the direction of Th17 cell-mediated inflammation and the phenotype of these cells (98–100). The physiological importance of SRC-1 and SRC-3 to Th17 cell-associated activity has been demonstrated by resistance to autoimmune pathogenesis in SRC-1 KO (98) or SRC-3 cKO (in CD4 cells) (99) EAE mice.

SCR-3 is the most dominant p160 family member in terms of the regulation of lymphoproliferation. As opposed to its proliferative role in various cancers, SRC-3 restrains the proliferation of lymphoid cells and HCSs (74, 103). SRC-3 is highly expressed in immature hematopoietic cells and is critical for their healthy development and function (103, 104). In mature lymphocytes, SRC-3 also is important for their proper functionality, as has been demonstrated by NK anti-tumor activity (107) and the suppressive capability of Tregs (104). SRC-1 and SRC-2 have opposing roles in determining the fate of CD4+ T cells with regard to the differentiation of these cells into Tregs: SRC-1 shifts the phenotype of naïve CD4+ T from an immunoregulatory to an inflammatory state, by promoting their differentiation into Th17 cells, rather than Tregs. SRC-2, on the other hand, stimulates naïve CD4+ T cells to differentiate into Tregs.

In summary, the SRC family members have a broad range of interactions with different components of the immune system. They can directly interact with factors that have primary role in the regulation of systemic immunity, such as NF-KB, and can also participate in fate determination of specific sub-sets of immune cells. The biological impact of the SRCs in immunity is highly diversified and context-dependent, hence it can be pro- or anti-inflammatory and take place under basal or stimulating conditions. The well-established and diverse functionality of the SRCs in immunity highlights their attractiveness as potential targets in autoimmune disorders and cancer.

Due to lack of a high-affinity ligand binding pocket and the fact that protein–protein interactions largely define their biological activity (7, 12, 110), the SRCs have been considered ‘undruggable’ targets (12, 111). Recently the challenge of identification of naturally occurring (112–114) and development of synthetic small molecule modulators (inhibitors and stimulators) (85, 86, 115–118) for the SRCs has been met, which opened an opportunity for new drug-treatment strategies in cancer, especially in therapy-resistant diseases (119). Considering the significant accumulation of data that establish the SRCs as important regulators of immunity and recent advances in the ability to target these proteins with small-molecules, present an opportunity for exploring new strategies in drug treatment in cancer and immune disorders. For example, two recent studies have shown that a small molecule stimulator and Small molecule inhibitor (SMI) of SRC-3 both can be used to modulate the immune environment for an achievement of better therapeutic results in two different pre-clinical mice models representing two different diseases: The use of a SRC-3 stimulator MCB-613 promoted the establishment of an anti-inflammatory environment that supported tissue repair post-MI, suggestively by suppressing MΦ–mediated inflammation signals and shifting the balance of MΦ population at the wound site from pro- to anti-inflammatory (86, 120). The diversity of the regulatory roles of SRC-2 and SRC-3 in MΦs offers an opportunity to further explore them as targets for therapeutic intervention on a context-dependent basis. Another study has demonstrated, using an immune-intact syngeneic mice model, that inhibition of SRC-3 by SMI SI-2 reshapes the cytokine milieu in the tumor site which drives the infiltration of cytotoxic immune cells, such as CD8 + and CD56 + (NK cells), into the tumor microenvironment (TME) and limits the number of immune suppressive FOXP3+ cells there, leading to enhanced anti-tumor immunity and suppression of tumor progression (118). The observation that SRC-3 inhibition controls the trafficking of Treg cells into the TME together with evidences that suggest the regulation of Treg biology by SRC-2 and SRC-3 (104, 108) creates an impetus for the targeting of the SRCs in Treg related pathologies that span from autoimmunity to cancer. In cancer, Tregs are central contributors to the immune -evasion ability of tumors, and their increased tumor infiltration is inversely correlated with clinical outcomes (121). Checkpoint immunotherapy that disrupts the immunosuppressive interactions between Tregs and effector cells and between Tregs and antigen presenting cells (APCs), achieved significant advancement in cancer treatment (122–124). Yet, immune checkpoint therapy has had limited success and is effective for only a few subtypes of cancer (125). In this regard, small molecule inhibitors that have been developed to suppress the oncogenic activity of the SRCs, can potentially fulfill a double-purposed therapeutic effect by targeting both cancer cells and Tregs at the same time to pursue an improvement in clinical outcomes of existing onco-immunotherapeutic methods.

The role of Th17 cells in autoimmune disorders is well established. Considering an unambiguous impact of SRC-1 and SRC-3 on the phenotype and function of Th17 cell and its pathophysiological consequences, provides a rationale for targeting these coactivators as a potential therapeutic approach for autoimmune disorders.

## Author contributions

YG wrote the first draft of the manuscript. All authors have made a substantial intellectual contribution to the work. All authors contributed to the article and approved the submitted version.
